# Empowerment of women and mental health promotion: a qualitative study in rural Maharashtra, India

**DOI:** 10.1186/1471-2458-7-225

**Published:** 2007-08-31

**Authors:** Michelle Kermode, Helen Herrman, Rajanikant Arole, Joshua White, Ramaswamy Premkumar, Vikram Patel

**Affiliations:** 1 Australian International Health Institute, University of Melbourne, Melbourne, Australia; 2 Comprehensive Rural Health Project, Jamkhed, Maharashtra, India; 3 St Vincent's Hospital, Melbourne, Australia; 4 Schieffelin Leprosy Research and Training Centre, Tamil Nadu, India; 5 London School Hygiene and Tropical Medicine, London, United Kingdom and Sangath Centre, Porvorim, Goa, India

## Abstract

**Background:**

The global burden of mental illness is high and opportunities for promoting mental health are neglected in most parts of the world. Many people affected by mental illness live in developing countries, where treatment and care options are limited. In this context, primary health care (PHC) programs can indirectly promote mental health by addressing its determinants i.e. by enhancing social unity, minimising discrimination and generating income opportunities. The objectives of this study were to: 1. Describe concepts of mental health and beliefs about determinants of mental health and illness among women involved with a PHC project in rural Maharashtra, India; 2. Identify perceived mental health problems in this community, specifically depression, suicide and violence, their perceived causes, and existing and potential community strategies to respond to them and; 3. Investigate the impact of the PHC program on individual and community factors associated with mental health

**Method:**

We undertook qualitative in-depth interviews with 32 women associated with the PHC project regarding: their concepts of mental health and its determinants; suicide, depression and violence; and the perceived impact of the PHC project on the determinants of mental health. The interviews were taped, transcribed, translated and thematically analysed.

**Results:**

Mental health and illness were understood by these women to be the product of cultural and socio-economic factors. Mental health was commonly conceptualised as an absence of stress and the commonest stressors were conflict with husbands and mother-in-laws, domestic violence and poverty. Links between empowerment of women through income generation and education, reduction of discrimination based on caste and sex, and promotion of individual and community mental health were recognised. However, mental health problems such as suicide and violence were well-described by participants.

**Conclusion:**

While it is essential that affordable, accessible, appropriate treatments and systems of referral and care are available for people with mental illness in developing country settings, the promotion of mental health by addressing its determinants is another potential strategy for reducing the burden of mental illness for individuals and communities in these settings.

## Background

Mental illness affects one in four people at some stage during their lives. It is estimated that 450 million people are experiencing mental illness at any one time, most of whom live in developing countries [1]. Mental illness is associated with high levels of health service utilization and associated costs, and in developing countries these costs are mostly paid for out-of-pocket. About one-quarter of patients using primary health care (PHC) services in developing countries have a mental illness [1]. Despite this substantial burden of disease and the availability of effective and affordable treatments, mental health care remains a neglected issue, especially in developing countries [1,2].

A range of factors influence the prevention, onset and course of mental illness including poverty, sex, age, disasters and conflicts, major physical diseases, and family and social environments [1,3]. Women are more susceptible than men to common mental disorders. Possible reasons for this include biological factors such as hormonal regulation; social factors such as the imposition of rigid traditional roles especially those that restrict women's personal liberty and agency and attribute lower social status to women; and exposure to domestic and sexual violence [1].

### Mental health and women in India

Depressed women in India often present with somatic rather than emotional symptoms [4,5]. Consequently, when they attend busy primary care settings with somatic complaints, the diagnosis of depression is often overlooked [6] and families incur unnecessary costs for healthcare consultations, investigations and treatments, and psychosocial interventions are not initiated.

Depressed women in India attribute their condition to a range of factors including poverty, traditional expectations of women's role, lack of affection and conflict with their husbands, widowhood and divorce, and difficulties providing dowries for their daughters [5,7]. Post-natal depression in India affects 11–23% of women, and is linked to poverty, antenatal psychiatric morbidity, poor marital and family relationships, lack of support, marital violence and birth of a girl child [8-10].

Women subjected to violence have a higher risk of mental illness including depression, anxiety and psychosomatic symptoms [11]. Violence against women in India is a common social problem. A household survey of 500 women in rural Maharashtra reported 23% had been beaten in the last six months. The husbands of 12% of these women had explicitly threatened to pour kerosene on them and set them alight [12].

### Mental health care in India

The National Mental Health Program, launched in 1982 and reviewed in 1995, sought to integrate mental health care with PHC [13]. It has had mixed success in achieving this goal and 90% of the rural population remain without access to mental health services [4,13,14]. Most care for people with mental illness is provided by the family. Many people remain untreated, and those families who do seek treatment will often turn to non-allopathic providers including practitioners of traditional medicine, religious healers, faith healers and astrologers.

### Mental health promotion

Mental health is more than the absence of mental illness and is defined by the World Health Organisation (WHO) as *a state of well-being in which the individual realises his or her own abilities, can cope with the normal stresses of life, can work productively and fruitfully, and is able to make a contribution to his or her community *[15]. A mental health promotion framework that represents a public health approach to mental illness prevention identifies the following as key social and economic determinants of community and individual mental health [16]:

**1. Social inclusion**: social relationships, involvement in group activities, civic engagement

**2. Freedom from discrimination and violence**: valuing diversity, physical security, self-determination and control over one's life

**3. Access to economic resources**: work, education, housing and money

Promoting mental health by addressing these determinants is a strategy for preventing mental illness, just as promoting exercise and a healthy diet prevents heart disease and diabetes.

### The Comprehensive Rural Health Project

The Comprehensive Rural Health Project (CRHP) was founded in 1970 and is located in a rural, drought-prone area of western Maharashtra. The CRHP is based on the principles of community-based PHC (equity, integration and empowerment), serves a population of approximately 250,000, with a strong focus on women. Interventions include income generation; agricultural and environmental programs; education; PHC, hospital and referral services; and rehabilitation for disabilities. The CRHP adopts a community development approach engaging with local people through groups including Farmer's Clubs, Mahila Mandal or women's groups, and adolescent girls' groups. The Mahila Mandal groups bring women together for social, health and educational reasons as well as income generation activities. Over three decades the CRHP has improved the health of the communities it serves [17,18]. Achievements include a substantial reduction in diseases such as leprosy, tuberculosis and malaria and a marked decline in infant mortality to less than 40/1000 live births (*cf *110/1000 for the state). The position of women in the society has improved and caste barriers have diminished [17,18].

At the heart of the Program are the Village Health Workers (VHWs) who are trained volunteers. The VHWs are often from low-caste or low-status groups such as single mothers or widows. Empowerment of individuals and communities involves creating opportunities for those who are powerless so that they can gain the experience and confidence needed to influence decisions that affect their lives [19]. Central to the empowerment process is enhancing individual competence and self-esteem which, in turn, increases perceptions of personal control and this has a direct positive effect on health outcomes [20]. Even though the aim of the CRHP has primarily been to improve physical health through the process of empowerment, many of the programs and activities actively address the social and economic determinants of mental health [18]. The links between the CRHP's interventions and the mental health promotion framework discussed above provide the rationale for the study described in this report. The study objectives are to:

1. Describe concepts of mental health and beliefs about determinants of mental health and illness among women involved with the CRHP

2. Identify perceived mental health problems, specifically depression, suicide and violence, their perceived causes, and existing and potential community strategies to respond to them

3. Assess the impact of the CRHP on individual and community factors associated with mental health

## Methods

### Study design and sampling

This qualitative study utilised the method of in-depth semi-structured interviews conducted with 32 women associated with CRHP. We interviewed 16 VHWs and 16 village women as we anticipated differences in perceptions and understandings of mental health between the two groups. During the course of a VHW meeting and four Mahila Mandal meetings at villages located close to CRHP, the purpose, nature and ethical aspects of the study were explained by members of the research team and group members were invited to participate.

### Data collection

A theme list was developed by the researchers to determine the direction and content of the interviews based on the study objectives and informed by the literature, and finalised with local staff to ensure that questions were appropriate for the context. The theme list was translated into Marathi and back-translated into English to ensure equivalence of the questions, and was piloted with women at CRHP. The appropriate Marathi words for concepts such as 'mental illness' and 'depression' were discussed extensively. This qualitative approach was considered appropriate for achieving the objectives of this exploratory study because the participants were unfamiliar with the concept of research, came from a culture with a strong oral tradition, and were mostly illiterate. The interview themes included concepts and determinants of mental health and illness; risk groups; examples of mental illness; depression; suicide; violence; interventions; and the impact of the CRHP. Five locally recruited interviewers included a nurse, two social workers and two VHWs. Their training covered ethical aspects of the study and the skills required for effective interviewing. The interviews were conducted in late 2004 in a private setting at CRHP. All were digitally recorded and lasted between 30 and 150 minutes.

### Data analysis

The interview transcripts were thematically analysed. This inductive approach involves systematically identifying themes and patterns within the data. The interview transcripts were coded largely according to the areas covered in the theme list. Following this, patterns that emerged within each theme were identified.

### Ethical issues

This study was approved by the Human Research Ethics Committee at the University of Melbourne, Australia, and reviewed by senior staff at CRHP for cultural appropriateness. Informed consent was obtained from all participants, participation was entirely voluntary and confidentiality assured.

## Results

The average age of the participants was 44 years (range 25–64). Most had been involved with the CRHP for a long time, with the average length of involvement for the VHWs being 18 years (range 5–31) and for the women from the Mahila Mandal groups 13 years (range 2–29). The views and experiences of the VHWs and the village women were very similar, so the findings from the two groups were analysed together.

### Concepts and determinants of mental health and illness

The participants described a mentally healthy woman as having no 'worries', 'tension' or 'pressures' in her life. She looks physically healthy, has a smiling face and her mind is 'open' and 'free', and she is 'bold' and 'confident'. Almost every participant mentioned that a mentally healthy woman has a harmonious home life, which is largely determined by the quality of her relationships with her husband and in-laws, especially her mother-in-law. Most of the women expressed this as an absence of negative relationships. Only a few mentioned the presence of positive relationships as important for women's mental health. Another important characteristic of a mentally healthy woman was financial security usually expressed as having enough money for food and clothing, and having a secure place to live. Some women commented that financial independence from their husbands was also important for women's mental health, i.e. women having their own source of income.

A mentally healthy woman was also characterised as having the freedom to move around and make her own decisions without having to defer to her husband. Some participants mentioned children as a positive influence on women's mental health: not only having children (especially sons), but also the children being educated, employed, healthy and well-behaved. Additionally, a few participants mentioned enough rain and good crops as important factors. The following quotes typify the responses.

She is very open-minded and she is quite bold with men and women as well. The conversation is confident. She seems to be free of any pressures or tensions. She always partakes in the village activities. She is willing to mediate when there is some quarrel. She is always very sympathetic and above all she is bold. She is capable of thinking. She is prepared for her own enhancement because of good health. She helps her husband in the house, listens to him and makes him listen to her. She is capable of making her own decisions about buying things for the children, opening bank accounts. She is respected in the household and also by the village members. Therefore she has got good mental health. She has no ill will towards anybody and she is always willing to help anybody. She has enough food and clothing. She has the capacity to think and manage the house. She has the ability to get on well with the family members... She is confident that she is no less than the menfolk. [VHW 03]

Such a woman does not have any worries. She feels free. She is carefree. Her mind is open. She is always happy as she has everything that she wants and she requires. She is quite contented financially and mentally. Her family life is happy. She is treated with love and concern by her husband and the mother-in-law... Her husband is not addicted. She is not ill-treated by her in-laws. Such things keep her in good health... She is financially independent. She has the power without anybody's restriction to make her own decisions regarding herself and her family at different occasions... If she is educated and employed she makes her own decisions. [VHW 05]

Mental illness was commonly conceptualised as mental tension or pressure and the determinants of mental illness for women were predominantly relational and economic, and generally the inverse of those associated with mental health. Essentially, if a woman has a husband and mother-in-law who do not treat her well, and her husband squanders their money on alcohol and gambling, then there will be conflict in the home and not enough money for food and clothing. The participants were of the view that due to these factors the woman would suffer from worries, pressure and tension, that is mental illness.

A cart has two wheels and they should move together. If one wheel pulls and the other drags then there is no mental health. Then to release the tension he finds the easy way of drinking and she will be nervous and depressed. Then how will she have good mental health? If he keeps relations with other women then how will she have good mental health? You see, if everything is normal then there is mental health. If husband and wife fight and quarrel then there is no mental health. [VHW 05]

Other determinants of mental illness for women included: having daughters and no sons, having too many children, being infertile, lacking the freedom to move around, no independent source of income, violence, poor crops and drought.

If a woman has five daughters and her husband is a drinker, she is greatly worried about the future of her daughters. She has many problems. For example, how to marry off these five daughters? How to arrange the dowry money? The daughters also get depressed. They too think and get worried about their future as their father's a drunkard. They lose interest in life. [VHW 05]

Participants were asked if they knew of anyone in their village with a mental health problem, and many recounted anecdotes of people behaving in ways that suggested mental illness including serious mental illness such as psychosis. In particular, they were asked about depression, suicide and violence, and each of these is discussed below.

### Perceptions of mental illness in the community

#### Depression

The participants were read a short story about a woman named Meena who was experiencing a constellation of symptoms that could well be depression.

Meena is 22 years old and was fine until six months ago when she began to feel tired all the time. She said that she was sad and had lost interest in life. Even her children and family didn't make her feel happy. She could not sleep, and she lost the taste for food, which she used to love. She even lost interest in cooking because she could not concentrate. Once she felt like ending her life.

When asked what sort of problem Meena has, most participants recognised depression as a possibility and projected onto the story a range of reasons for her depression that were congruent with their understanding of the determinants of mental health and illness described above. When asked if they knew of similar cases, some of the participants readily described women from their own villages who had experienced what may well have been an episode of depression.

Participant: (P): Take the example of my close relative, a girl whose husband died and so she was brought to my home. Everybody was after getting her remarried. After the remarriage she became mentally derailed. In due course she became pregnant and things got worse. She used to behave strangely. She had no sense of personal hygiene.

Interviewer (I): What do you think was the cause of all this?

P: It seems that she was remarried against her will. After the delivery of the child, she did not care for it at all. In fact, she was positively cruel to him. Then the doctors came and saw her and she was advised to take some tablets for a period of three months. Very gradually she improved and started behaving slightly better. After sometime she was almost totally cured. She started caring for the baby. [VHW 04]

#### Suicide

Every participant knew of at least one person who had either attempted or completed suicide. The methods of suicide involved self-immolation, hanging, poisoning and jumping in the well. Two-thirds of the stories involved a woman. Some of people were well known to the participant, and in a few cases participants described their own suicide attempts.

My own daughter committed suicide. I was a widow at an early age. I raised the little daughter and she was studying in the sixth class. There was pressure on us to get her married off at a tender age and I could not resist the pressure. So she was married off, at the age of about eleven. Initially they looked after her well and at an early age she became pregnant. As this happened her other sister-in-law became jealous because she had no child... The sister-in-law ill-treated her, snatched the baby boy from her and asked her to get out. My girl became desperate and hanged herself. [MM 10]

P: One day my husband drank a lot and the whole night he did not allow me to sleep. Whenever I tried to sleep, he took away the bedding from me and was saying bad words. That day I went to another farm to work and I was very tired but my husband did not allow me to sleep. Then I just pretended that I was sleeping. I put all my children to sleep. My husband was also sleeping. I was very angry, so I took a rope and tried to hang myself... I just went inside the house, closed the doors and I was about to hang myself. But somehow my husband came to know so he called some other people. They were all trying to break open the house. Some were trying to break the doors whereas some were trying to take off the roof. There was a lot of confusion and noise. In that noise my children also got up from sleep. By that time, by the grace of God, I was also calmed down. I did not feel like committing suicide. Then the people told me to think about the children. They advised me if I do that then who will look after the children. Then I gave up that idea.

I: Why did you attempt to commit suicide?

P: My husband drinks a lot and after drinking he troubles me, beats me like anything. One day he broke my finger due to this I've got a lot of pain. This was the reason I attempted to commit suicide. [MM 02]

The participants were asked what, in their opinion, were the reasons for the identified cases of suicide, and the reasons for suicide more generally. The commonest reason given for actual suicides was family conflict between husbands and wives or between parents and children. Others included financial problems, alcoholism, AIDS, exam failure, jealousy, drought, and for women specifically, infertility and the birth of daughters or failure to give birth to a son. The most common reasons given for suicide more generally was conflict in the family, especially between the woman and her husband or between the mother-in-law and daughter-in-law. In these cases the woman chooses suicide as a form of escape.

If the mother-in-law tortures her daughter-in-law... And if you are from a Maratha family, you can't just go back to your parent's home... Then the anger becomes uncontrollable, it becomes too much, and when it is too much it becomes unbearable to the brain. That anger meets the brain like a fever. One who is calm can think a little longer. The person who is not so calm loses control of her mind. [MM 05]

Other reasons for suicide included anger/impulsivity, money problems, dowry demands, and pregnancy out of wedlock.

#### Violence

Anecdotes describing violence, including domestic, physical and sexual violence, were relatively commonplace. The violence usually involved a man being violent to a woman, most often a husband to a wife. There were also descriptions of wives being violent to husbands, mothers-in-law being violent to daughters-in-law, fathers being violent to children, and (adult) children being violent to elderly parents.

P: Some young girls have had rape attempted on them. Some girls try to save themselves by shouting and gaining the attention of other people for help. Other helpless girls put up with it for fear of getting a bad name in the society. (They) just keep quiet about it.

I: What happens to the person who attempts this sort of violence?

P: Invariably nothing happens. He goes scot-free

I: How does the violence affect the subject?

P: She gets mentally disturbed. She becomes depressed. She doesn't want to share the information with anybody for fear of being discredited. [MM 09]

The participants linked much of the violence to alcohol use by the perpetrator. Other factors were identified as triggers for violence including infertility, birth of daughters, inability to meet dowry demands, arguments over money and jealousy.

One day [a man in the village] took an axe and started running after his wife. He was blaming her for giving him a girl child. He did not listen that a girl child is like a boy child. He said that he'll push the girl in the well because if he is childless he can remarry. I told his wife to come and stay with me for some time and asked another woman to take care of the daughter. I brought his wife to my house and reassured her. I stood at my door and told her husband, don't dare to enter my house. If he tried, I'd catch hold of his collar and push him out. I'd tell him that he has no right to come in. He was under the influence of alcohol. I warned him not to enter my house after consuming alcohol. If he came, I'd give him a thrashing. I took the help of two women, in case he came, we could attend to him. But he did not turn up. [VHW 01]

Some women recounted stories of their own husbands being violent, and a few alleged that their husbands had attempted or threatened to murder them. The link between being a victim of violence and having poor mental health was well-recognised by many of the participants.

When a person is done injustice [meaning violence] then the victim person loses his mental balance. He takes a lot of tension and sometimes behaves like a mad person. And if she is a woman then she doesn't bathe, doesn't comb her hair, and is not taking care of anything in the family. She always thinks about whatever happens to her then she takes a lot of tension. [MM 02]

Widowed women were identified as a risk group for violence, especially sexual violence. Mistreatment of the elderly was noted by some participants, as was the vulnerability of adolescent girls.

Alcohol abuse was mentioned frequently as contributing to mental illness, especially the effect of a man's alcoholism on his wife's and children's mental state. No-one referred to alcohol abuse as a mental health issue, or to the fact that mental illness may contribute to alcohol abuse. The impact of alcohol abuse on the mental health of individuals and families is encapsulated in the following quote:

We have, of course, a relative whose husband is a drunkard. He makes her life difficult. For alcohol he sold all his belongings. One day he beat her severely. She was so fed up that she attempted suicide by hanging. We rescued her and took her to the hospital in town. She improved and went back to the house but her condition is the same, her husband is the same and there is no reprieve. [MM 10]

### Impact of the CRHP on the determinants of mental health

All participants shared the view that the interventions of the CRHP had influenced the lives of individuals and communities in ways that were highly valued. Many of the women gave heartfelt personal testimonies regarding the impact of the Project on their own lives, especially the women who had taken on the role of VHWs.

Since I have joined CRHP, I am educating my children. Previously I was doing a labourer's job. Now my two sons are at Pune. They have purchased a small plot and have built a bungalow and are owners of two cars... They are now running a grocery shop and STD booth. Thus I have progressed a lot since I came into contact with you here. My husband, though uneducated, cooperates with me. He has no objections even though all this is my enterprise... Now my husband listens to me and I am satisfied. [MM 04]

There were multiple and consistent examples of the Project's positive impact on the determinants of mental health and well-being, especially in relation to addressing stigma and discrimination, and fostering economic participation. However, despite the transformative influence of CRHP on the lives of the people it serves, a considerable burden of mental illness was nevertheless communicated in the course of the interviews, as is evident from the descriptions above.

#### Social inclusion

Participants described the ways in which the CRHP promotes social relationships, involvement in groups, and civic engagement. The success of the Mahila Mandal and adolescent girls groups were commonly acknowledged, and the extent to which civic engagement has been encouraged by fostering community responsibility for and ownership of programs was evident. The importance of reaching out to help poorer members of the community was also recognised.

#### Freedom from discrimination and violence

The promotion of social tolerance was frequently described, and its success in reducing discrimination based on sex, caste, disability and illness was highlighted by all participants. The breakdown of caste barriers was most commonly attributed to two strategies. Firstly, the CRHP sited communal wells close to the homes of 'untouchable' and low-caste members of the village, so that higher caste families were forced to draw water from wells that had been 'polluted' by untouchable and low-caste contact. Previously, wells had been preferentially sited next to houses belonging to high-caste members of the village, and untouchable and low-caste families could only get access to water when others would draw it for them because they were not allowed to approach the well themselves. The second strategy involved the provision of supplemental feeding for all infants and children together during times of drought.

When in the beginning we came together, we had caste discrimination among us, but it is no more there. We say that though we are different, our soul is the same. We all are children of the same god so there should not be any difference among us. Our food habits, our way of dressing in clothes or colours may be different, but we are the same. So this is how we came to a common understanding. This happened due to education, which we got through this project. [MM 01]

Anecdotes describing greater self-determination for women in the community as a direct consequence of CRHP's interventions were frequently described.

We bring the girls to attend the meetings at Jamkhed. There they hear, see and learn many useful things. Now the girls have become clever and bold due to the knowledge and the information they received at Jamkhed... They learn tailoring, embroidering, and earn money. They also do some small business such as selling vegetables in the market and become economically independent even when they are at their parent's house. Then in future after marriage they carry the same work and will not depend on their husbands. [VHW 11]

P: My children also have the information. My son knows cooking. I want to say that I haven't made any difference between my son and daughter while rearing them up. My relatives also know. They have taken pictures of my son while he was cooking. My husband also helps out in my work. There's a lot of change.

I: How do you look after your son and daughter?

P: Equally. I usually give less work to my daughter. I tell my son sweetly, 'Dear you do this work, your sister is busy studying'. My son is in eleventh class and my daughter is in ninth class. If I had not had all this CRHP training, I wouldn't have educated my children up to this higher level. I might have stopped their education at fourth class only. I persuade the girls in the community to educate themselves. On 15^th ^August (Independence Day) and 26^th ^January (Republic Day) I give a speech in our school that educating girls will benefit them. [VHW 01]

Some participants described reduced levels of discrimination due to illnesses such as leprosy as a consequence of participation in CRHP activities. Safety from the threat of physical violence was mentioned by a few participants, but not nearly as commonly as the promotion of social tolerance and self-determination for women.

### Access to economic resources

Most participants reflected that women's mental health was enhanced by interventions that promote their capacity to earn income independent from their husbands. They mentioned a range of strategies for achieving this, including promoting literacy and numeracy, the formation of self-help (micro-credit) groups, training women in marketable skills, and providing seed funding/loans for businesses.

We now have women who go to the bank to deposit earnings there, and if the bank manager tries to pressurize them, then they give fitting replies. Previously they were not allowed to move out of the house but now they can manage such affairs. They are now properly trained. [MM 04]

I can see a lot of changes that have taken place in my life. Previously we were not given money at all but now I am handling the money matters. I go to Jamkhed to sell vegetables and the money I get, I keep with me. I even do all the shopping and marketing for the family. The other day I sold some eggs for Rs.150 and when I told my husband about this, he told me to keep the money with me. This is a major change I can see in my life. I am able to handle the money... Before I was not allowed to go anywhere but now I have freedom and I also use that freedom in a responsible manner. Whatever money I get, I use that money properly... I also understand that if we have money in our hand then people talk to us or give respect to us – otherwise there is no respect for us. So there is a lot of difference that I can see in my life. [VHW 05]

## Discussion

The key findings of this qualitative study among women from rural Maharashtra participating in a well-established PHC project are summarised as follows: The women viewed the determinants of mental health and illness as predominantly cultural and socio-economic; mental health was commonly conceptualised as an absence of stress and the most commonly identified stressors were conflict with husbands and mother-in-laws, domestic violence and poverty; women's mental health and women's empowerment were inextricably linked for these participants; and the CRHP's activities were perceived to be effectively addressing the determinants of mental health, but a range of mental health problems were also highlighted.

India is characterised by a range of diverse cultural and philosophical systems, mixed in recent years with Western modes of thinking, making it difficult to identify a uniform Indian paradigm of mind and mental health [21]. Furthermore, acceptance of allopathic forms of treatment does not necessarily equate with acceptance of the bio-medical perspectives that underpin such treatments, and many explanations for mental illness can be found [22]. Depressive illness is understood by some using a Western bio-medical framework, and by others using more traditional concepts such as Aryuveda that attribute illness, including mental illness, to an imbalance of humours and/or heat and cold. Supernatural, astrological and religious explanations are also common including karma, evil-eye and spirit possession [13,23,24].

The women in our study had a concept of mental health and depressive illness that is characterised by the presence or absence of 'pressure', 'worries' or 'tension', which roughly equates to the Western concept of stress. They understood mental health and depressive illness to be the product of relational and economic factors that contribute directly to the presence or absence of stress in women's lives, and consequently their mental health and well-being. Although our findings are similar to those of other authors who investigated explanatory models for depressive illness among Indian women [4,5,10], it is interesting that no participant in this study mentioned religious, super-natural, karmic or astrological factors. The absence of these explanatory models may have been due to participants' unwillingness to disclose them as the CRHP's health education program discourages such beliefs. On the other hand, no participant suggested that an imbalance of humours, heat and cold, or chemicals within the brain or body are the cause of mental illness, even though explanatory models such as these are compatible with traditional and allopathic systems of healing supported by the Project.

The women in this study attributed the development of mental illness exclusively to strained relationships and deprivation. Follow-up research is indicated to explore how they might explain the advent of mental illness in a woman who is living in comfort and has a good relationship with her husband and in-laws. Additionally, it is not clear that their explanations for more serious mental illnesses such as schizophrenia would be the same as those outlined above. Supernatural explanations may be more relevant in such cases.

The relational and economic factors identified as determinants of mental health for women were primarily external to the women themselves, and often beyond their control. Essentially, if a woman is lucky enough to be married to a man who does not drink or gamble, remains faithful and earns some money, and the mother-in-law does not harass her, and she has sons, then she will have good mental health. As Indian village women are usually unable to earn their own money, cannot influence the sex of their child, and are rarely able to choose their husbands and mothers-in-law, the perceived determinants of their mental health and well-being are located largely outside their control. A constellation of reasons including socio-cultural expectations, their own sense of duty and obligation, and financial dependence on others make it difficult for women in conflicted family situations to escape. For many this has a negative impact on their mental health.

The women identified a range of ways in which the CRHP assisted them to gain a measure of control over their own lives. They frequently acknowledged that the opportunity to independently earn money resulted in a range of positive changes at both the individual and family levels. In turn, this was seen to have a direct and desirable impact on mental health. Increased freedom of movement and greater participation in decision-making were linked to economic participation, and these were also seen as important for women's sense of competence and control, and consequently for their mental health.

Descriptions of suicide were commonplace in this study and a range of reasons for suicide were provided including family conflict, financial difficulties, alcoholism, violence and failure to produce a son. The pattern of suicide in developing countries such as India is different from that observed in developed countries. For example, the male to female ratio for successful suicide in developed countries averages 3:1 whereas in India it is 1.4:1 [25]. The most common means of suicide in India are pesticide poisoning, hanging, self-immolation and drowning, and this was reflected in the findings of this study. The descriptions of attempted and completed suicide in most of the interviews highlight the seriousness of this problem in rural Indian communities, which is well-recognised elsewhere in the country [26,27]. There is a significant problem of young married women committing suicide, often in relation to dowry demands and conflict with the husband and parents-in-law [28,29]. In south India suicides accounted for 8–12% of total deaths, and women aged 15–24 years were more likely to commit suicide than males of the same age (164/100,000 *vs *96/100,000) [26].

A strategy for effectively integrating mental health into PHC includes promoting mental health by addressing the key determinants of social inclusion, freedom from discrimination and violence, and economic participation. From the perspective of the study participants, CRHP interventions are successfully addressing the determinants of mental health and have positively changed the lives of many people, especially women. However, suicide, violence, alcohol abuse and other mental health problems remain major concerns for women, their families and communities. Extending the reach of the Project so that it more actively addresses the inter-linked problems of violence and alcohol abuse would likely benefit both men and women.

When interpreting these findings a number of limitations should be kept in mind, some of which are inherent to research conducted in a resource-poor setting. A number of biases are possible as the sample was self-selecting: women whose lives had been affected in some way by a mental health problem or women who felt particularly indebted to the CRHP may have been more likely to participate. The possibility of social acceptability bias influencing some responses also needs to be considered as the interviewers were not independent of the Project. The sample size is not large, but a larger sample size is unlikely to affect the findings as data saturation was rapidly reached.

It is difficult to assess to what extent the perceived changes in attitudes and behaviours reported by these women are attributable to the influence of the CRHP and how much is due to wider social changes in India itself. However, the reported benefits of CRHP on the well-being of women specifically and the community generally [17] suggest that the women connected to this Project may be in a unique situation. Views of women from other areas of rural Maharashtra could well be different. The findings reported here reflect participants' perceptions, which may or may not accord with reality. Further systematic investigation is needed to quantify the prevalence of and risk factors for mental illness, suicide and violence in the Jamkhed community.

## Conclusion

**Figure 1:**
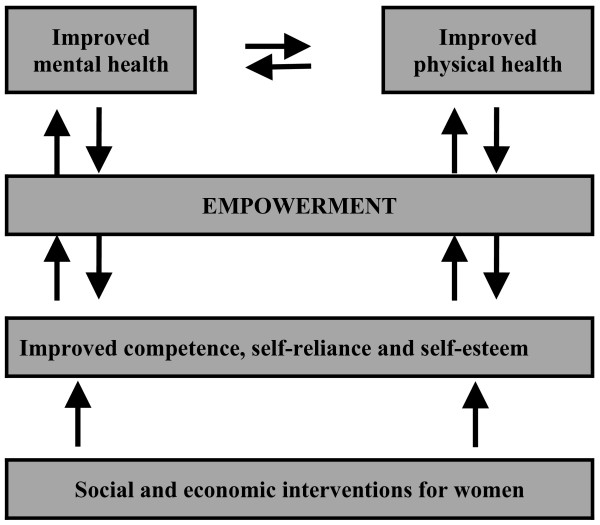
A model for the relationship between social and economic interventions for women, empowerment and health.

The findings of this study invite the possibility that mental health and empowerment are closely aligned concepts, and that their realisation potentially has shared and mutually reinforcing pathways (Figure 1). Therefore, development interventions to strengthen communities may also promote mental health and well-being. More formal assessment of the relationship between these two concepts is warranted.

Even though effective mental health promotion is a useful strategy for minimising the impact of mental illness, it remains essential that affordable, accessible, appropriate treatments are available and that reliable systems of referral are established for people with serious mental illness that cannot be managed at the PHC level. Increasing community awareness in relation to detection of and response to mental illness by providing relevant training at the PHC level (e.g. to the VHWs at CRHP) is a feasible and potentially beneficial intervention. Finally, improvements in mental health for people in developing country settings requires health systems strengthening as well as attention to other development issues such as gender and power relationships, access to economic resources and human rights.

## Abbreviations

PHC, Primary health care; WHO, World Health Organisation; CRHP, Comprehensive Primary Health Care Project; VHWs, Village Health Workers

## Competing interests

The author(s) declare that they have no competing interests.

## Authors' contributions

All authors made a substantial contribution to conception and design of the study and were involved in drafting and reviewing the manuscript. MK, JW, RA, PK and VP contributed to data acquisition. MK, HH and VP contributed to analysis and interpretation of the data. All authors have read and approved the final manuscript.

## Pre-publication history

The pre-publication history for this paper can be accessed here:


